# Supplementary material for: Dendrite fragmentation induced by massive-like δ–γ transformation in Fe–C alloys
Source: Nat Commun. 2019 Jul 18;10:3183. doi: 10.1038/s41467-019-11079-y (PMC6639379; doi:10.1038/s41467-019-11079-y)
Supplement: Supplementary file 1 — Description of Additional Supplementary Files [file 41467_2019_11079_MOESM1_ESM.docx]

Description of Additional Supplementary Files

**Supplementary Movie 1:** Fragmentation at austenite grain boundary after the massive-like transformation.

**Supplementary Movie 2:** Multiple fragmentation of dendrite arms at the δ – γ interface via a reverse peritectic reaction.
